# Molecular Mechanisms of Gonadotropin-Releasing Hormone Signaling: Integrating Cyclic Nucleotides into the Network

**DOI:** 10.3389/fendo.2013.00180

**Published:** 2013-11-20

**Authors:** Rebecca M. Perrett, Craig A. McArdle

**Affiliations:** ^1^Laboratories for Integrative Neuroscience and Endocrinology, School of Clinical Sciences, University of Bristol, Bristol, UK

**Keywords:** GnRH, G-proteins, phospholipase C, adenylyl cyclase, guanylyl cyclase, ERK, PACAP, natriuretic peptide

## Abstract

Gonadotropin-releasing hormone (GnRH) is the primary regulator of mammalian reproductive function in both males and females. It acts via G-protein coupled receptors on gonadotropes to stimulate synthesis and secretion of the gonadotropin hormones luteinizing hormone and follicle-stimulating hormone. These receptors couple primarily via G-proteins of the G_q/ll_ family, driving activation of phospholipases C and mediating GnRH effects on gonadotropin synthesis and secretion. There is also good evidence that GnRH causes activation of other heterotrimeric G-proteins (G_s_ and G_i_) with consequent effects on cyclic AMP production, as well as for effects on the soluble and particulate guanylyl cyclases that generate cGMP. Here we provide an overview of these pathways. We emphasize mechanisms underpinning pulsatile hormone signaling and the possible interplay of GnRH and autocrine or paracrine regulatory mechanisms in control of cyclic nucleotide signaling.

## Gonadotropin-Releasing Hormone Receptors and Effectors

Gonadotropin-releasing hormone (GnRH) (pGlu-His-Trp-Ser-Tyr-Gly-Leu-Arg-Pro-Gly-NH_2_), also known as luteinizing hormone-releasing hormone (LHRH) or GnRH I, is a hypothalamic neuropeptide that mediates central control of reproduction in both males and females. It is synthesized in hypothalamic neurons and secreted from the hypothalamus into the hypophyseal portal circulation in pulses which are most often of a few minutes duration. It acts via GnRH receptors (GnRHRs) on gonadotropes within the anterior pituitary, stimulating the synthesis and secretion of luteinizing hormone (LH) and follicle-stimulating hormone (FSH), thereby controlling gametogenesis and steroidogenesis (1–6). GnRH is absolutely required for reproduction as demonstrated by mutation of the genes encoding GnRH or its receptor (7–9).

Molecular phylogeny of GnRH ligands shows that there are three distinct forms, GnRH-I, GnRH-II, and GnRH-III that arose from a common origin which predates vertebrates (10). Most vertebrate classes have GnRH-I and GnRH-II (1, 3, 11), whereas GnRH-III has only been found in teleosts (12–23). Interestingly, the GnRH-I sequence has diverged in the vertebrate lineage, whereas the sequences of GnRH-II and GnRH-III are completely conserved across vertebrates (3, 10, 24).

## Clinical Uses

Gonadotropin-releasing hormone analogs are used clinically, either to mimic its stimulatory effects, such as the treatment of infertility with pulsatile administration of a natural sequence of GnRH to induce ovulation or spermatogenesis (3, 25), or to block its effects. The latter can be achieved either using GnRH antagonists (1–6, 26, 27), or, paradoxically, with sustained exposure to GnRH (or metabolically stable GnRH agonists), which causes stimulation followed by desensitization of GnRHR-mediated gonadotropin secretion (3, 25). In both cases blockade or desensitization of GnRHR-mediated gonadotropin secretion ultimately reduce circulating levels of gonadotropins and gonadal steroids, and in this fashion GnRH analogs can be used to treat sex hormone-dependent neoplasms such as those of the prostate, ovary, endometrium, or mammary glands (1–6, 28).

## Gonadotropin-Releasing Hormone

GnRH receptors belong to the rhodopsin-like G-protein coupled receptor (GPCR) family, and are thus characterized by a seven trans-membrane α helical domain structure (3, 29, 30). GnRHRs can be classified into three groups based on sequence homology. All of the cloned mammalian GnRHRs are in groups I or II (3, 24) and the type I GnRHRs of humans, rats, mice, pigs, sheep, and horses have >80% amino acid sequence homology (31). Except in certain primate species, notably the marmoset, rhesus, and green monkey, the type I receptor is the functional and predominant form expressed in the mammalian gonadotrope, and in some species it is also expressed in extra-pituitary tissues including breast, gonads, prostate, and uterus (32, 33). This extra-pituitary expression is also evident in numerous cancers, including breast, prostate and ovary, and on *in vitro* or *in vivo* tumor models GnRH analogs or cytotoxic derivatives show promise as anti-proliferative and/or pro-apoptotic agents (34–40).

**Figure 1:**
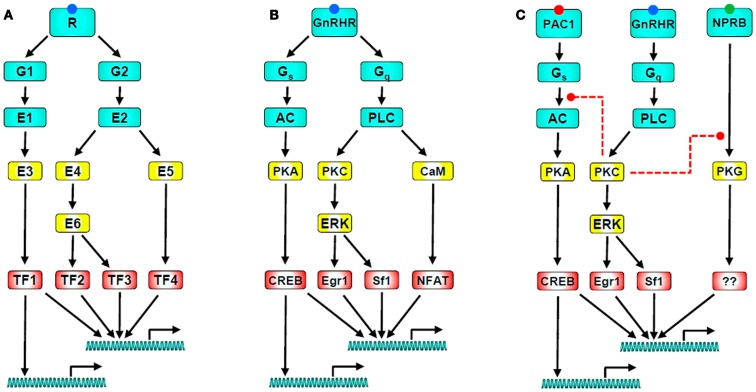
**GnRH receptor signaling networks**. **(A)** Illustrates a generic signaling network in which a GPCR activates two heterotrimeric G-proteins (G1 and G2) which activate their cognate effectors (E1 and E2). These directly or indirectly activate down-stream effectors that influence a range of target proteins including transcription factors (TF1-4). The transcription factors then act (typically in combination) to influence expression of numerous target genes. Note that the network has multiple sites for divergence and convergence. **(B)** Shows a GnRH signaling network with the same architecture; The GnRHR activates Gs and Gq leading to activation of adenylyl cyclase (AC) and phospholipase C (PLC). AC generates cAMP, stimulating PKA which activates the transcription factor CREB. PLC leads to activation of PKC, driving activation of ERK and of ERK-dependent transcription via Sf-1 and Egr-1. It also elevates the cytoplasmic Ca^2+^ concentration, driving activation of calmodulin and its targets, including calcineurin which leads to activation of the Ca^2+^-dependent transcription factor NFAT. This cartoon is clearly a vast oversimplification as important effectors (including calmodulin-dependent kinases, JNK, p38, and nitric oxide synthase) are not included. Perhaps more importantly, it also excludes signal dynamics and heterologous regulation, both of which are important for control of gonadotropes. A simple example of the latter is given in **(C)** which includes the PAC1 receptor as a mediator of PACAP-stimulated AC activation, and the NPRB receptor as a mediator of CNP-stimulated cGMP accumulation and consequent protein kinase G (PKG) activation. GnRH can cause PKC-mediated inhibition of PACAP-stimulated cAMP accumulation and of CNP-stimulated cGMP accumulation (as indicated by the dashed red lines), raising the possibility that its predominant effect is actually inhibition of these pathways in gonadotropes exposed to autocrine or paracrine stimulation of PAC1 and NPRB. Finally, when considering signal dynamics, it is important to recognize: (a) that GnRH is secreted in pulses, (b) that the responses illustrated have distinct kinetics, (c) that the kinetics of convergent pathways are important determinants of GnRH pulse frequency-response relationships, and (d) that GnRH influences the expression of many genes encoding components of the GnRHR signaling pathways, with transcription-dependent feedback loops supporting an adaptive signaling network.

In common with many other GPCRs, GnRHRs of gonadotropes and gonadotrope-lineage cells act primarily via Gα_q/11_ to activate phospholipase C (PLC), thus elevating cytoplasmic [Ca^2+^] and activating protein kinase C (PKC) isozymes, both of which are important for GnRHR-mediated effects on gonadotropin synthesis and secretion (Figure 1) (2–6, 29, 31, 41–46). The mammalian type I GnRHR is a structurally and functionally unique member of the GPCR family in that it lacks an intracellular cytoplasmic C-terminal tail (3, 47, 48). For many GPCRs, the C-tail plays a key role in desensitization and trafficking (49, 50). The C-terminal tail of typical GPCRs is phosphorylated on Ser and Thr residues following activation, generating a docking site for non-visual arrestins (arrestins 2 and 3) that prevent G-protein activation, a process termed homologous receptor desensitization. The phosphorylated tails also act as adapters targeting the desensitized receptors for internalization, a process that can lead to receptor down-regulation, or recycling and resensitization (49, 51). The absence of a C-terminal tail would therefore imply an inability of the type I mammalian GnRHR to undergo agonist-induced phosphorylation or bind arrestins, with relatively slow internalization and resistance to rapid desensitization, all of which have been confirmed experimentally (51–61). In addition, fusing the C-terminal of various GPCRs to the type I mammalian GnRHR causes rapid desensitization and internalization (51, 54, 62–65). Both the rat and human GnRHR internalize in a clathrin-dependent manner, and colocalize with transferrin, which is internalized via clathrin-coated structures (54, 56, 59). The rat GnRHR internalizes in a dynamin dependent manner (64), whereas the human internalizes independently of dynamin (47). Contrastingly, upon activation type II GnRHRs do undergo rapid agonist-induced phosphorylation, recruit arrestins, and internalize via clathrin-coated pits (47). The requirement for arrestins and dynamin is species specific (3, 66, 67), but the presence of the C-terminal tail is crucial for rapid agonist-induced internalization (60).

Non-mammalian GnRHRs may also activate extracellular signal-regulated kinase (ERK) in an arrestin-mediated manner. Arrestins can act as adaptors for signaling molecules, for example cRaf1 and the ERK mitogen-activated protein kinase (MAPK), both of which can bind to MAPK/ERK kinase (MEK), and could therefore participate in MAPK activation (68–72). Arrestin-mediated ERK signaling appears specific for non-mammalian GnRHRs; in cells either expressing a mouse type I or a *Xenopus laevis* GnRHR, both caused G-protein-dependent ERK activation but arrestin-mediated ERK activation was only seen with the C-tail expressing *Xenopus* GnRHR (73, 74). An interesting possibility is that the C-terminal tail was lost through evolution because the GnRH pulses that gonadotropes are exposed to would be too short to evoke desensitization of a C-tailed receptor, such that there was no selective advantage for retention of the structure. Alternatively, its loss may be related to the pre-ovulatory gonadotropin surge that is driven by GnRH pulses of increasing frequency and possibly also increased amplitude and a failure to return to basal levels between frequent pulses. Receptor desensitization under such conditions could conceivably prevent the pre-ovulatory gonadotropin surge, providing a positive adaptive advantage for loss of the rapid homologous receptor desensitization mechanism.

## Heterotrimeric G-Protein Coupling

In pituitary gonadotropes, GnRHR signaling is primarily mediated by Gα_q/11_ subunits, although GnRHR coupling to Gα_i_ and Gα_s_, as well as Gα_q/11_ have also been reported (75–78). Agonist binding is associated with GTP loading of Gα_q/11_, which activates phospholipase C β (PLCβ), elaborating the second messengers inositol 1,4,5-trisphosphate (IP_3_), and diacylglycerol (DAG). IP_3_ mediates Ca^2+^ release from intracellular stores, and DAG causes activation of PKC isozymes (Figure 1). A more sustained rise of intracellular Ca^2+^ occurs via the opening of L-type voltage gated channels and subsequent Ca^2+^ influx (1–6, 79). Progressively increasing GnRH concentrations cause three different Ca^2+^ responses, subthresholds, baseline oscillations, and biphasic responses (80–82). The initial spike phase is due to mobilization of Ca^2+^ from intracellular stores, which is involved in early GnRH-stimulated LH release (83), whereas the plateau corresponds to Ca^2+^ entry through voltage-dependent Ca^2+^ channels. The oscillatory responses are generated through a cytoplasmic Ca^2+^ oscillator model (84). Rapid effects of GnRH on exocytotic gonadotropin secretion are mediated by elevation of cytoplasmic Ca^2+^ and modulated by activation of PKC. These signaling intermediates, and effectors including calmodulin and calmodulin-dependent protein kinases (CaMKs), also mediate chronic effects of GnRH on gene expression.

Gonadotropin-releasing hormone effects on gonadotropin synthesis are largely mediated through stimulation of MAPK cascades, particularly the ERK pathway (Figure 1) (85), which is PKC dependent in αT3-1 and LβT2 gonadotrope-derived cells (79). PKC and ERK mediate the transcriptional effects of GnRH on the common α-gonadotropin subunit (αGSU) (86–89), as well as LHβ (90–93) and FSHβ (93–96) subunits. However, there are conflicting reports that GnRH-mediated LHβ (88) or αGSU expression (97, 98) are independent of ERK and mediated solely by Ca^2+^. There are also gender specific difference in mice with pituitary specific knockout of ERK1 and 2; females are infertile due to LH deficiency, and ERKs may play a partial role in FSHβ transcription in these mice, however male reproductive function was normal (99).

In addition to activation of ERK, GnRH can activate the JNK (c-Jun N-terminal kinase), p38, and ERK5 (also known as Big MAPK; BMK) cascades in different cell models with varying kinetics. GnRH stimulates JNK activity in rat pituitaries, αT3-1 and LβT2 cells (79, 100, 101). JNK has been reported to be involved in transcription of the αGSU subunit (102, 103), and the LHβ and FSHβ subunits (94, 101, 104, 105). JNK-mediated LHβ transcription is independent of PKC in LβT2 (90, 105) and COS (106) cells, with conflicting reports for PKC involvement in αT3-1 cells (85, 107). GnRH also activates p38 in rat pituitaries, αT3-1 and LβT2 cells (91, 94, 103, 104, 108). A role for p38 in gonadotropin subunit transcription is controversial, with no effect being reported on LHβ, FSHβ, and αGSU subunits (91, 103, 104), although an effect on FSHβ transcription in LβT2 cells was reported by others (94, 108). GnRH has also been shown to activate ERK5 and stimulate FSHβ transcription in LβT2 cells (109).

GnRH receptors can also activate a number of other pathways in pituitary gonadotropes, including the adenylyl cyclase (AC)/cyclic adenosine monophosphate (cAMP)/protein kinase A (PKA) pathway (79, 110, 111). Borgeat et al. (112) demonstrated that GnRH-stimulated cAMP production in the rat pituitary, which was later confirmed by Naor et al. (113). GnRH also stimulates cAMP production in LβT2 cells (77, 111, 114), and several heterologous systems including HeLa, GH_3_, and COS-7 cells (115–117). However, this was not replicated in αT3-1 cells or in later studies using rat pituitaries (45, 118, 119). The coupling mechanism between the GnRHR and the cAMP pathway has yet to be elucidated. The GnRHR has been reported to couple to Gα_s_ in rat pituitary cells (76), and activate cAMP production via Gα_s_ recruitment (77). However, in αT3-1 cells the GnRHR exclusively coupled to Gα_q/11_ (120), and activation of Ca^2+^/calmodulin sensitive AC isoforms independent of Gα_s_ was proposed as the mechanism of GnRHR-induced cAMP elevation. In addition, the PKC δ and ε isoforms were reported to mediate cAMP elevation by GnRH via activation of AC5 and 7 in LβT2 cells (111). However, a more recent study using a biosensor to monitor cAMP mobilization in living cells has demonstrated that GnRH increases cAMP production in αT3-1 cells, and that the GnRHR directly interacts with SET protein, which inhibits receptor coupling to calcium and increases coupling to the cAMP pathway, possibly by interfering with Gα_q/11_ binding to the GnRHR (121). In LβT2 and mouse pituitary cells, GnRH activates AMP-activated protein kinase (AMPK) via multiple pathways involving Egr-1 and JNK, and AMPK inhibition suppresses GnRH-stimulated LHβ transcription (122).

Gonadotropin promoter subunits contain cAMP response elements (CREs) and this provides a mechanism by which the cAMP/PKA pathway might activate gonadotropin subunit transcription (Figure 1). αT3-1 cells demonstrate a four- to fivefold increase in phospho-CREB (CRE-binding protein) in response to GnRH (123). cAMP stimulates transcription of the mouse, rat, and human αGSU genes (124, 125), and a cAMP analog increased αGSU mRNA levels in rat pituitary cells, but not that of LHβ or FSHβ (126). However, it appears the MAPK cascade, rather than the cAMP pathway, is responsible for gonadotropin promoter CRE activation (90, 100, 107, 127, 128). Here it is important to recognize that CREB can be regulated by MAPKs, CaMKs, and PKC as well as by PKA (129). c-Jun and ATF-2, which are known substrates of JNK, were shown to bind to the CRE domain of the αGSU promoter (130). GnRH phosphorylates ATF-2 via p38 and JNK, and upon phosphorylation ATF-2 binds the CRE element within the c-Jun proximal promoter and interacts with nuclear factor Y. Functional ATF-2 is necessary for both GnRH-mediated induction of c-Jun and FSHβ (131). In addition, GnRH treatment increases expression of ATF-3, which is recruited along with c-Jun and c-Fos to CREs on the αGSU promoter, and GnRH-induced αGSU gene expression was completely abolished upon mutation of these CREs (132). MAPK signaling and ATF-3 CRE binding are essential for secretogranin II promoter activation by GnRH (133).

GnRH receptors activate a large number of important signaling pathways, notably, they mediate activation of phospholipases A2 and D as well as PLC (41). GnRH-mediated intracellular Ca^2+^ mobilization, acting through calmodulin, also activates kinases such as Ca^2+^/CaMKs, phosphatases such as calcineurin and transcription factors including nuclear factors of activated T-cells (NFATs) (79, 134). GnRHR-induced elevation of intracellular Ca^2+^ also activates the nitric oxide synthase (NOS I) cascade (NOS I/NO/soluble guanylate cyclase) resulting in a rapid increase of cGMP (135–137). However there is no evidence that cGMP is involved in GnRH-induced gonadotropin synthesis or secretion (135) (see subsequent section). GnRH also activates the Wnt/β-catenin signaling pathway as well as diacylglycerol kinase, proline rich tyrosine kinase-2, and inhibition of glycogen synthase kinase (1, 6, 41, 138–140).

In addition to directly activating a number of intracellular signaling pathways, in some models GnRHRs can also cause a PKC-dependent proteolytic release of membrane bound epidermal growth factor (EGF) receptor ligands, thereby activating EGF receptors (1, 41), whereas in others GnRHRs induce protein phosphatases that apparently inhibit the trophic effects of EGF (78). Moreover, in HEK293 cells stably expressing the type I GnRHR, GnRH causes cytoskeletal remodeling, which correlates with significant increases in the tyrosine phosphorylation status of a series of cytoskeletal associated proteins, including focal adhesion kinase (FAK), c-Src, and ERKs (139). ERK activation is dependent on formation of a complex with FAK and c-Src at focal adhesion complexes, and induction of the cell remodeling event is mediated by activation of the monomeric G-protein Rac, revealing a novel monomeric G-protein-mediated pathway for GnRHR signaling (139).

## Pulsatile GnRH Signaling

Gonadotropin-releasing hormone is released from hypothalamic neurons as pulses causing pulsatile gonadotropin release (141, 142), and these pulses are essential for normal reproduction; constant GnRH suppresses LH and FSH secretion, and this can be restored by pulsatile administration (143). GnRH pulses are typically a few minutes in duration, every 30–120 min according to the species.

It is well established that the frequency of such pulses is extremely variable. For example, GnRH pulse frequency varies over the menstrual cycle with pulses on average every 6 h in mid- to late-luteal phases and every 90 min during follicular and early luteal phases (144). Low or intermediate pulse frequencies (pulses every 30–120 min) cause a greater increase in expression of rodent LHβ, FSHβ, and the GnRHR as compared to high frequencies (pulses every 8–30 min) or sustained stimulation (145–151). The expression of αGSU does not exhibit this bell-shaped frequency-response relationship and is maximally stimulated by high pulse frequencies or continuous stimulation (148, 149, 152, 153).

The ability of the gonadotrope to interpret varying pulses of GnRH has been the focus of much research, given that differential responses of LH and FSH occur with varying GnRH pulse frequency, both *in vivo* and *in vitro*. In ovariectomized rhesus monkeys bearing hypothalamic lesions which reduced circulating LH and FSH to undetectable levels, hourly pulses of GnRH favored LH secretion over FSH, whereas pulses every 3 h favored FSH secretion and caused a decline in LH levels (154). Additional *in vivo* studies with GnRH deficient men recapitulated this observation (155, 156), as do *in vitro* studies using pituitary cultures (145–151, 157), with intermediate pulse intervals (30 min–1 h) favoring LHβ transcription and low frequencies (every 3 h) that of FSHβ. Although most work on GnRHR signaling has involved sustained stimulation, similar signaling mechanism appear to be involved in response to pulsatile stimulation, including activation a number of key effectors including Gα_q/11_, Gα_s_, and Gα_i_ (41, 79, 158). Downstream of Gα_q/11_, the Ca^2+^/calmodulin/calcineurin/NFAT and Raf/MEK/ERK signaling modules are both activated (159, 160) (see below), and gonadotrope ERK has been shown to be essential for reproduction (99) consistent with its role as an effector of pulsatile GnRHR activation *in vivo*.

The mechanisms by which gonadotropes decode GnRH pulse frequency are largely unknown, despite the fact that this frequency-encoded signal is crucial for the physiology and therapeutic manipulation of the reproductive system (2, 27, 99, 109, 159–162). A key feature of this system is that maximal responses to some effects of GnRH occur with sub-maximal pulse frequencies. In essence this means that there is a bell-shaped frequency-response curve for some effects of GnRH, behavior that has been termed “genuine frequency decoding” (163) to distinguish it from the simpler situation where increasing pulse frequencies elicit increasing responses up to the maximal pulse frequency (i.e., constant stimulation). The bell-shaped frequency-response curve is thought to require more complex systems involving feed-forward or feedback regulation (163) and is exemplified by the non-monotonic relationships seen for effects of GnRH on LHβ or FSHβ expression (as measured using luciferase reporters). However the nature of the negative feedback loop is unclear. It could lie at the level of upstream components of the GnRHR cascade; GnRH causes down-regulation of cell surface GnRHRs (164) and a recent mathematical model of GnRH signaling predicts desensitization due to down-regulation of cell surface GnRHRs, which is more pronounced at higher pulse frequency (165). It cannot however be due to rapid homologous receptor desensitization as type I mammalian GnRHRs do not show this behavior (58). Alternatively, transcription-dependent negative feedback on upstream inputs could occur at high GnRH pulse frequency. This could include GnRHR-mediated induction of regulator of G-protein signaling (RGS)-2 which displays GTPase activating protein activity and is known to inhibit Gα_q/11_ signaling (166, 167), direct interaction of the GnRHR with SET protein which may inhibit Gα_q/11_ binding (121), or induction of MAPK phosphatases (MKPs) which would modulate GnRHR-mediated ERK signaling (109). GnRH also causes down-regulation of IP_3_ receptors (168, 169), and induces expression of calmodulin-dependent small G-protein Kir/Gem (kinase-inducible Ras-like protein/GTP binding protein over-expressed in skeletal muscle), which is known to inhibit Ca^2+^ channels (145). Finally, the feedback or feed-forward regulatory loops could lie further downstream, within the nucleus. Low pulse frequency causes transient Egr-1 expression, causing expression of co-repressor Nab-2, thus inhibiting LHβ expression. With high GnRH pulse frequencies there is a more sustained increase in Egr-1, which increases LHβ transcription by quenching Nab-2 (162). However, neither upregulation of Nab-1 and Nab-2, or differential expression of Egr-1, were observed *in vivo* (101). The proteasome has been proposed to play a role in cyclical hormonal responses, by targeting transcription factors for degradation and thus freeing the promoter to enable it to respond to the next pulse of hormone (170) GnRH-mediated LHβ gene expression is dependent on protein degradation via the proteasome, and Egr-1 and SF-1, two key transcription factors for LHβ, are targets of the ubiquitin-proteasome system (171). Targeting transcription factors for degradation would promote gonadotrope sensitivity, allowing more rapid transcriptional responses to changes in GnRH concentration.

There appears to be selective interplay of factors at the *Fshb* promoter according to pulse frequency: mutation of a CRE site within the FSHβ promoter resulted in loss of preferential GnRH stimulation at low pulse frequencies (161), and low pulse frequencies stimulated PKA activity and levels of phospho-CREB compared to high pulse frequencies (172). AP-1 family members FOS and JUN positively regulate the *Fshb* promoter and are induced at low GnRH pulse frequencies, whereas SKIL and TGIF1 corepressors negatively regulate the *Fshb* promoter, and are induced at higher frequencies (173), along with ICER, which antagonizes the stimulatory action of CREB to attenuate FSHβ transcription (161). As well as inducing c-Fos expression, low GnRH pulse frequencies act via the ERK1/2 pathway to cause c-Fos phosphorylation, which extends its half-life, thereby enhancing FSHβ transcription (174).

In order to test for upstream feedback mechanisms during pulsatile GnRH signaling, we have used live cell imaging reporters including an NFAT1c-emerald fluorescent protein (NFAT-EFP) and ERK2-GFP (159, 175). Nuclear translocation of NFAT-EFP provides a readout for elevation of intracellular Ca^2+^ because the Ca^2+^/calmodulin-dependent activation of calcineurin causes dephosphorylation of cytoplasmic NFAT that exposes a nuclear localization sequence (176). Similarly, activation of ERK causes it to be released from cytoplasmic scaffolds and facilitates protein-protein interaction necessary for nuclear entry, such that the redistribution of ERK2-GFP from the cytoplasm to the nucleus can provide a readout for activation of the Raf/MEK/ERK cascade. In HeLa cells transduced to express type I GnRHR, pulsatile GnRH caused rapid NFAT-EFP and ERK2-GFP nuclear translocation, but with markedly different response kinetics. With 5 min GnRH pulses, ERK2-GFP translocated rapidly to and from the nucleus and the nuclear:cytoplasmic (N:C) ERK2-GFP measure returned to basal values between stimuli, whereas the N:C NFAT-EFP response was slower in onset and offset so that at high pulse frequency the response had not returned to the pre-stimulation value before a subsequent stimulus was added (159, 175). This led to “saw-tooth” or cumulative response, thought to increase signal efficiency with pulsatile stimuli (177). Irrespective of these differences, there was no evidence for desensitization of responses to pulsatile GnRH using these readouts (175) and maximal responses were seen at maximal GnRH pulse frequency. In contrast, maximal effects were seen with sub-maximal pulse frequencies when luciferase reporters containing LHβ or FSHβ promoters were used as experimental readouts. Thus, the bell-shaped frequency-response curve or “genuine frequency decoding” of GnRH pulses is not a specific feature of gonadotropes and can occur in the absence of the negative feedback previously thought to explain it.

The studies outlined above focused on the Ca^2+^/calmodulin/calcineurin/NFAT and Raf/MEK/ERK pathways because both mediate transcriptional effects of GnRH and both decode pulse frequency in other models (178–183). The promoter regions of gonadotropin genes contain response elements likely to mediate the effects of ERK (i.e., Egr-1 sites) and NFAT (181), and the Raf/MEK/ERK and Ca^2+^/calmodulin/calcineurin/NFAT cascades are known to act as co-dependent modules in other systems, notably in the control of cardiac myocyte proliferation where ERK and NFAT converge on composite AP-1/NFAT response elements in a number of genes (180, 182). In spite of this, the empirical data provided no explanation for the observed bell-shaped frequency-response relationships so a mathematical approach was taken to explore this further.

We have developed a mathematical model for GnRHR signaling based on a series of ordinary differential equations describing GnRHR occupancy and activation and downstream effectors (27). This differs from earlier models (109, 163, 165, 184–186) in that it incorporates Ca^2+^/calmodulin/calcineurin/NFAT and Raf/MEK/ERK modules, includes cellular compartmentalization (i.e., nuclear versus cytoplasm) and importantly, lacks upstream negative feedback. This model accurately predicts wet-lab data for activation and nuclear translocation of ERK2-GFP and NFAT-EFP as validated by modeling responses to GnRH pulses at a range of concentrations and frequencies, and therefore these two could be used as inputs to the transcriptome. Using this model we considered the possibility that two transcription factors (TF1 and TF2) act at distinct sites on a common gene promoter named gonadotropin subunit (GSU), a generic term used because this is likely the case for the αGSU, LHβ, and FSHβ gonadotropin subunit genes, as it is for many other ERK and NFAT target genes (178–183). We tested three distinct logic gates for the nature of the action of TF1 and TF2 at the promoter (27). The first is a co-operative GATE that in biological terms could reflect the action of one TF to mediate the interaction between the other TF and the cells transcriptional machinery, or alternatively, the requirement of physical interaction between the two TFs to orientate distant promoter sites and bring them to close proximity for transcription activation. The second is the AND GATE in which both TFs are needed for transcription activation but there is no functional interaction between them, and the third is the OR GATE where either or both TFs can drive transcription but there is again no functional interaction between the two.

This model predicted bell-shaped frequency-response relationships when two TFs act co-operatively. The characteristic feature of maximal response at sub-maximal frequency was never seen with the AND-gate or with the OR-gate, and this behavior was predicted in the absence of negative feedback which is often assumed to underlie it. This modeling also implied that GnRH pulse frequency-response relationship may be plastic, as varying rate constants for transcription factor activation and inactivation, or varying balance of signaling via NFAT and ERK-dependent transcription factors, influenced the pulse frequency predicted to give a maximal response (27).

The importance of the modeling outlined above is that a bell-shaped frequency-response relationship is predicted to be an emergent feature of co-operative and convergent signaling of two signaling pathways. It requires that the pathways have distinct response kinetics and occurs in spite of the fact that individual pathways and pathway components cannot generate this complex relationship (27). It does not, however, establish that the bell-shaped frequency-response relationships seen for transcriptional effects of GnRH are necessarily mediated by convergence of NFAT and ERK-dependent transcription factors. In reality, multiple pathways converge to mediate GnRH effects on transcription, with the relative importance and integration of these inputs being specific for the promoter/enhancer studies (187). In this context, it is of interest that a recent study explored the contribution of Gα_s_ and Gα_q_ signaling for pulsatile GnRH signaling. In this work FRET reporters were used as live cell readouts for activation of the PKA and PLC signaling pathways via the endogenous mouse GnRHR of LβT2 cells (188). This revealed that pulses of GnRH cause pulses of cAMP elevation and PKA activation that are rapid and transient, and do not show measurable desensitization from pulse to pulse (188). This is in accord with the lack of upstream adaptive mechanisms seen with live cell imaging of ERK2-GFP and NFAT-EFP (above). However, the FRET readouts for elevation of Ca^2+^ and DAG (measures for PLC activation) desensitized rapidly from one pulse to the next (188). This raises the intriguing possibility that co-operative convergent effects of the Gα_s_ and Gα_q_ pathways could mediate GnRH pulse frequency decoding and also that the balance of PKA to PLC signaling varies through a series of GnRH pulses. However, the PLC data are puzzling as desensitization of PLC responses with repeated pulses would be expected to be coupled with desensitization of downstream responses, yet repeat pulses of GnRH can elicit comparable effects on cytoplasmic Ca^2+^ (189, 190), on gonadotropin secretion (5, 191), and on NFAT-EFP translocation (above). It is also unclear why GnRH-mediated PLC activation would desensitize with repeat GnRH pulses, when PLC-mediated [^3^H]IP accumulation does not show desensitization with up to 60 min of sustained stimulation (47, 53, 57, 192). Using siRNA and bacterial toxins to specifically perturb individual G proteins in LβT2 cells, Choi et al. demonstrated that FSHβ expression was dependent on Gα_q_, whereas Gα_s_-mediated LHβ transcription and suppressed that of FSHβ (193). Inhibinα was identified as a Gα_s_ dependent GnRH-induced autocrine/paracrine factor which suppresses FSHβ transcription. Its transcriptional profile contrasts with that of FSHβ, being induced by high pulse frequencies, and therefore its absence at low pulse frequencies may explain the preference for FSHβ transcription.

## Autocrine and Paracrine Regulation of Gonadotropes

Given the crucial role of GnRH in reproduction, it is not surprising that most work on gonadotrope cell signaling has focused on its mode of action. However, gonadotropes are receptive to various other extracellular stimuli, including the gonadal steroids estrogen, progesterone, and testosterone, which as well as acting centrally to influence GnRH secretion, also act directly on the pituitary to modulate GnRH effects on gonadotropes. In addition to GnRH, gonadotropes are targets for a large number of GPCR-activating ligands (194). These include oxytocin, endothelin-1, galanin, β-endorphin, neuropeptide Y, nucleotides, and pituitary adenylyl cyclase activating polypeptide (PACAP), a highly conserved member of the vasoactive peptide (VIP)-secretin-glucagon peptide superfamily.

Here we highlight some additional signaling pathways key to cyclic nucleotide signaling in the gonadotrope.

## Pituitary Adenylyl Cyclase Activating Polypeptide

Pituitary adenylyl cyclase activating polypeptide was originally isolated from sheep hypothalamic extracts based on its ability to stimulate cAMP production by rat pituitary cell cultures (195). It is widely distributed in the nervous, immune, gastrointestinal, cardiac, and endocrine systems (195, 196). Two isoforms have been identified, a 38 amino acid form (PACAP38) and C-terminally truncated 27 amino acid form (PACAP27), with PACAP38 accounting for 90% of the protein in most tissues (194–198). The PACAP peptides have 68% amino acid homology with VIP but are 1000 times more potent in their ability to stimulate cAMP (196).

Three receptors are activated by PACAP; VPAC_1_, and VPAC_2_ which have similar affinity for VIP and PACAP, and PAC_1_, which is highly selective for PACAP (197–200). PAC_1_ receptors have the potential to couple directly to both Gα_s_ and Gα_q/11_ and exist as multiple splice variants due to alternative splicing of two exons in the third intracellular loop (hip and hop) and are named null (neither hip nor hop), hip, hop1, hop2, hiphop1, and hiphop2 (194, 198, 200–202). Early work showed that (for most PAC_1_ variants) PACAP38 and PACAP27 had comparable potency for stimulation of cAMP production, whereas PACAP38 was much more potent than PACAP27 for stimulation of IP accumulation (203).

Within the anterior pituitary, the major secretory cells and folliculo-stellate cells all express at least one type of PACAP receptor (200, 204). Various PAC_1_ receptor forms predominate in the rat pituitary and gonadotrope cell lines (205), and in these cells PACAP activates PAC_1_, causing a Gα_s_-mediated stimulation of cAMP production and a Gα_q/11_-mediated increase in [Ca^2+^]_i_ (194, 197, 200, 206–211). PACAP regulates gonadotropin secretion and expression of signature genes in gonadotropes either acting alone, or by modulating GnRH effects (194, 197, 200, 210, 212–218). PACAP can act alone or synergistically with GnRH to stimulate LH and FSH production (216, 219), although the effect of PACAP on LH secretion is modest compared to that of GnRH (215). Low pulse frequencies of GnRH promote PACAP and PAC_1_R expression compared to high pulse frequencies (220). In LβT2 cells, high frequencies of PACAP pulses increase LHβ transcription, whereas low frequencies promote that of FSHβ (as seen with GnRH pulses) (221). In addition PACAP and PAC_1_R expression increase with lower frequencies of PACAP pulses (221, 222). The action of GnRH in the regulation of gonadotropin subunit expression is enhanced by the presence of PAC_1_Rs (223). At present the mechanisms by which PACAP and its receptor contribute to FSHβ and LHβ expression are unknown, it may act to increase GnRHR expression via a cAMP mediated pathway (224).

Pituitary adenylyl cyclase activating polypeptide increases follistatin expression by gonadotropes and folliculo-stellate cells (211, 225, 226), and therefore may modulate activin signaling in the pituitary (197). PAC_1_ receptor activation causes much greater elevation of cAMP than GnRH does in gonadotrope-derived cell lines (200, 208, 209), and GnRH actually causes a PKC-mediated inhibition of PAC_1_-mediated cAMP elevation (208, 227). Therefore, if gonadotropes are exposed to stimulatory concentrations of (local or hormonal) PACAP *in vivo*, GnRH pulses could actually inhibit rather than stimulate cAMP production (208).

## Natruiretic Peptides, Nitric Oxide, and Guanylyl Cyclases

The natriuretic peptides atrial-, B-type, and C-type natriuretic peptides (ANP, BNP, and CNP respectively) act via cell surface guanylyl cyclase containing receptors to stimulate cGMP accumulation, which causes activation of protein kinase G (PKG) and cyclic nucleotide gated ion channels (Figure 1) (199). These are single trans-membrane enzymes which are thought to act as homodimers (199, 228). There are three subtypes of receptor, NPRA (GC-A) which has high affinity for ANP and BNP, NPRB (GC-B), which is selective for CNP, and the NPRC (GC-C) receptor which binds all three peptides and acts predominantly as a clearance receptor (229). The effects of ANP and BNP on hemodynamic and cardiovascular regulation are well documented (229, 230). The physiological roles of CNP are less clear, although a critical role in endochondral ossification is evident (231). CNP is expressed in LH positive cells of the anterior pituitary (232, 233), and female mice with either the CNP (*Nppc*) or GC-B (*Nprb*) genes deleted are infertile (231, 234).

CNP stimulates cGMP accumulation in GnRH neurons (235), pituitary gonadotropes (236), and endocrine cells of the testis, ovaries, placenta, and uterus (237–244), implying widespread roles of CNP in the hypothalamo-pituitary–gonadal (HPG) axis. In gonadotrope-derived cell lines, CNP activates the αGSU promoter (233), however it has no effect on LH secretion or GnRH-stimulated LH secretion (228, 233, 245). GnRH causes rapid PKC-mediated inhibition of CNP-stimulated cGMP accumulation in αT3-1 cells (228, 236), and may stimulate CNP expression, by transcriptional regulation of the *Nppc* gene (233). However, little is known about the actions of cGMP in the pituitary, so the physiological relevance of pituitary CNP/NPRA signaling remains unknown.

Gonadotropes also express the enzyme responsible for the generation of nitric oxide (NO), NO synthase (NOS) (246). The NOS enzyme family is composed of three major isoforms, neuronal NOS (nNOS), inducible NOS (iNOS), and endothelial NOS (eNOS). These enzymes convert l-arginine to l-citrulline, producing NO, an important signaling molecule involved in a variety of physiological and pathological processes (247). It exerts physiological effects by activation of soluble guanylyl cyclases to generate cGMP (248). nNOS and eNOS are expressed constitutively and activated by Ca^2+^/calmodulin, whereas iNOS is Ca^2+^-independent (129, 194). In the anterior pituitary, nNOS has been specifically detected in the folliculo-stellate cells and gonadotropes (137, 249). GnRH stimulates the activity and expression of nNOS in gonadotropes (136, 137, 250) and this likely explains the increase in nNOS expression and activity observed at proestrous (136). GnRH may activate the nNOS promoter via cAMP-dependent activation of a CRE in the GnRH-responsive region of the nNOS promoter (251, 252). Alternatively it may act via SF-1, which acts on a nuclear hormone receptor binding site on the nNOS promoter in pituitary gonadotropes to stimulate transcription (253).

Nitric oxide itself inhibits GnRH-stimulated LH secretion, with the NOS inhibitor MeArg markedly potentiating GnRH-induced LH secretion, and the NO donor SNP significantly reducing it (246, 254, 255). GnRH, LH, and FSH release are decreased in chronic NO deficiency (256, 257), and in humans treatment with an NOS inhibitor can reduce GnRH-stimulated LH and FSH release (258). The effects of NO on gonadotropin secretion remain rather controversial [see Ref. (194) for discussion of stimulatory and inhibitory effects]. Intriguingly, NO donors stimulate LH and FSH release in a cGMP-independent manner (254, 259) implying that these effects reflect regulation of NO targets other than soluble guanylyl cyclases.

## Conclusion

Type I mammalian GnRHRs of pituitary gonadotropes signal primarily via G_q/11_. Uniquely, they have no C-terminal tail and therefore do not elicit the C-tail dependent and heterotrimeric G-protein independent signaling seen with many other GPCRs. These features could ensure that the type I mammalian GnRHR of pituitary gonadotropes (e.g., the receptors that mediate central control of reproduction in humans) faithfully transduce the portal blood GnRH signal into PLC activation in the target cells, and this could arguably confer selective advantage by (i.e., facilitating the pre-ovulatory gonadotropin surge). Nevertheless, there is ample evidence that GnRHRs can activate other heterotrimeric G-proteins and that they do so in a cell context-dependent manner (44, 77, 111, 112, 115, 120, 208, 260–262). Notably, they apparently activate G_i_ in some hormone-dependent cancer cell models and activate G_q/11_, G_s_, and G_i_ in GT1-7 neurons. Early work in primary cultures of pituitary cells revealed that GnRH increases cAMP production (112, 113) but this would not necessarily reflect G_s_ activation and could even involve regulated cAMP production in cells other than gonadotropes. Subsequent work revealed little or no effect of GnRH on cAMP production in the gonadotrope-derived αT3-1 cell line (120, 208, 216) as opposed to the stimulatory effects seen in the more mature LβT2 gonadotrope cell line (77, 111). Such studies do not really address the fundamental question of physiological role. Thus, although it is well established that PLC-mediated effects on cytoplasmic Ca^2+^ and PKC influence exocytotic secretion of gonadotropins and transcriptional effects of GnRH, the relevance of GnRH effects on cAMP (and cGMP) production are much less clear. In this review we have highlighted two areas that may prove important in resolving this issue. The first is that paracrine or autocrine mechanisms exist for regulation of cyclic nucleotide production. Notably, PACAP has pronounced effects on cAMP production in gonadotropes and gonadotrope-derived cell lines, and the possibility exists that the modest stimulatory effects of GnRH pale into insignificance in gonadotropes exposed to PACAP. The second is that GnRH is secreted in pulses and very little is known about signaling of pulsatile GnRH via anything other than G_q/11_. Here, a key feature is that maximal effects of GnRH are often elicited at sub-maximal GnRH pulse frequency and mathematical modeling has revealed that such non-monotonic frequency-response curves could reflect co-operative activity of two (or more) convergent signaling pathways. This was explored for the Ca^2+^/calmodulin/calcineurin/NFAT and Raf/MEK/ERK pathways but the same logic could equally apply to either (or both) of these pathways acting together with the G_s_/AC/cAMP/PKA pathway. In this regard it is of interest that GnRHR activation actually reduces PACAP-stimulated cAMP production and CNP-stimulated cGMP production in αT3-1 cells (208, 236) raising the question of whether GnRH pulses are stimulatory or inhibitory for these pathways *in vivo*. Clearly, a great deal is yet to be learned about cyclic nucleotide signaling in gonadotropes and how the signaling network integrates inputs via PLC, AC and GC.

## Conflict of Interest Statement

The authors declare that the research was conducted in the absence of any commercial or financial relationships that could be construed as a potential conflict of interest.

## Abbreviations

Ca^2+^, calcium; cAMP, cyclic adenosine 3′,5′-monophosphate; EFP, emerald fluorescent protein; EGF, epidermal growth factor; ERK, extracellular signal-regulated kinase, here used to denote ERK1 and ERK2; FAK, focal adhesion kinase; FSH, follicle-stimulating hormone; GFP, green fluorescent protein; GnRH, gonadotropin-releasing hormone also known as GnRH I; GnRHR II, gonadotropin-releasing hormone II, also known as chicken GnRH; GnRHR, GnRH receptor; GPCR, G-protein coupled receptor; GRK, G-protein coupled receptor kinase; IP, inositol phosphate; JNK, c-Jun N-terminal kinase; LH, luteinizing hormone; MAPK, mitogen-activated protein kinase; MEK, MAPK/ERK kinase; NFAT, nuclear factor of activated T-cells; NO, nitric oxide; PKC, protein kinase C; PLC, phospholipase C.
